# Wanted a Good Hospital Advocate!

**Published:** 1888-11-17

**Authors:** Arthur W. Blake

**Affiliations:** Hon. Sec. Gorleston Cottage Hospital


### WANTED A GOOD HOSPITAL ADVOCATE!

WE intend next month holding a public meeting on behalf of this institution. Can you give me the name of a gentleman, a first-class speaker, an authority on the matter of Cottage Hospitals, who would come down here and advocate our cause. We want funds wherewith to build, as we have had a plot of land presented to us. Of course we are prepared to pay any reasonable expense attending the visit of anyone who would come. ARTHUR W. BLAKE, Hon. Sec.

Gorleston Cottage Hospital.

